# Investigating nutrient biomarkers of healthy brain aging: a multimodal brain imaging study

**DOI:** 10.1038/s41514-024-00150-8

**Published:** 2024-05-21

**Authors:** Christopher E. Zwilling, Jisheng Wu, Aron K. Barbey

**Affiliations:** 1 Department of Psychology, University of Illinois, Urbana, IL USA; 2 Beckman Institute for Advanced Science and Technology, University of Illinois, Urbana, IL USA; 3 Decision Neuroscience Laboratory, University of Nebraska-Lincoln, Lincoln, NE USA; 4 Center for Brain, Biology, and Behavior, University of Nebraska-Lincoln, Lincoln, NE USA; 5 Department of Psychology, University of Nebraska-Lincoln, Lincoln, NE USA; 6 Department of Bioengineering, University of Illinois, Urbana, IL USA

**Keywords:** Cognitive ageing, Biomarkers

## Abstract

The emerging field of Nutritional Cognitive Neuroscience aims to uncover specific foods and nutrients that promote healthy brain aging. Central to this effort is the discovery of nutrient profiles that can be targeted in nutritional interventions designed to promote brain health with respect to multimodal neuroimaging measures of brain structure, function, and metabolism. The present study therefore conducted one of the largest and most comprehensive nutrient biomarker studies examining multimodal neuroimaging measures of brain health within a sample of 100 older adults. To assess brain health, a comprehensive battery of well-established cognitive and brain imaging measures was administered, along with 13 blood-based biomarkers of diet and nutrition. The findings of this study revealed distinct patterns of aging, categorized into two phenotypes of brain health based on hierarchical clustering. One phenotype demonstrated an accelerated rate of aging, while the other exhibited slower-than-expected aging. A t-test analysis of dietary biomarkers that distinguished these phenotypes revealed a nutrient profile with higher concentrations of specific fatty acids, antioxidants, and vitamins. Study participants with this nutrient profile demonstrated better cognitive scores and delayed brain aging, as determined by a t-test of the means. Notably, participant characteristics such as demographics, fitness levels, and anthropometrics did not account for the observed differences in brain aging. Therefore, the nutrient pattern identified by the present study motivates the design of neuroscience-guided dietary interventions to promote healthy brain aging.

## Introduction

Accumulating evidence in Nutritional Cognitive Neuroscience indicates that diet and nutrition may benefit the aging brain (for a review, see ref. ^1^). A recent review of the literature surveyed 52 studies comprising more than 21,000 participants and found that dietary markers of the Mediterranean Diet were associated with healthy brain aging, as measured by MRI indices of structural and functional connectivity^2^. Despite the promise of these findings, questions remain about the causal effects of diet and nutrition on brain health and their role in age-related neurobiological decline; for example, whether elements of the Mediterranean Diet, such as polyunsaturated fatty acids (PUFAs), may limit the reduction in white matter volume with age. The potential benefits of the Mediterranean Diet may result from its focus on nutrient classes that have known functional relationships with the brain. For example, fatty acids, including monounsaturated, polyunsaturated, and saturated fatty acids, are necessary for structural brain integrity and development, cellular energy metabolism, and neurotransmission and neuromodulation^3^. Indeed, randomized controlled trials (RCTs) examining the effects of fatty acids on brain health typically observe improvements in brain function, white matter integrity, and gray matter volume^4–7^. Notably, however, RCTs that investigate the effects of fatty acids on cognitive performance (without additional measures of brain health) demonstrate mixed results, with positive^8^ or null findings^9^. In addition to fatty acids, the Mediterranean Diet includes antioxidants (i.e., vitamins, flavonoids, and carotenoids), which are known to reduce oxidative stress and therefore to benefit brain health^10,11^. RCTs examining the effects of antioxidants on the aging brain demonstrate benefits in cerebral blood flow and for measures of functional brain connectivity (e.g., functional brain network integration^12^). Evidence further suggests that antioxidants may have favorable effects on episodic memory, although these findings do not extend to all forms of memory affected by aging^7,13^

More broadly, a large association study of ~75,000 participants revealed that greater consumption of antioxidants was associated with a lower chance of developing subjective cognitive impairment in late life^14^. Finally, research also suggests that choline, an essential nutrient that promotes structural brain integrity, cellular energy metabolism, and neurotransmission, may improve multiple facets of cognition in older adults^15^. Taken together, these findings suggest that nutrition may support and enhance cognitive function and brain health, especially in healthy older adults.

The potential for nutritional interventions to promote healthy brain function is particularly significant given the well-established effects of aging on cognitive performance and brain health^16–21^. Senescence is accompanied by age-related neurodegeneration in gray and white matter structures and an increase in ventricular space^22^. White matter fiber integrity declines with age, as indexed by decreased fractional anisotropy and increases in axial, radial, and mean diffusivity^23^. Concentrations of metabolic markers of neuronal integrity, measured by magnetic resonance spectroscopy (MRS), also decline with age^22^. Advancing age is associated with smaller cerebral volume, likely due to cortical neuronal degeneration and synaptic density reduction, in addition to reduced cortical thickness and surface area^24,25^. The observed changes in the aging brain are also known to affect cognitive function, producing declines in cognitive control, fluid intelligence, processing speed, and memory^21,26–29^.

Age-related changes in brain health are known to vary within the population, reflecting individual differences in the onset, duration, and severity of age-related neurological symptoms^19,30,31^. Thus, chronological age alone does not fully explain the complex trajectory of brain health in late life. Indeed, recent evidence demonstrates that although structural MRI measures can predict chronological age, there are often deviations in the predicted and observed aging trajectory, such that accelerated aging results in a brain that is older than expected, whereas delayed aging results in a brain that is younger than predicted^32,33^.

Although the literature on healthy aging has identified important risk factors that accelerate brain aging, much less is known about preventative factors that reduce the severity of neurobiological disease in late life^34^. Our research therefore sought to identify nutrient biomarker patterns that are associated with Accelerated versus Delayed Brain Aging, with an interest in guiding the development of nutritional interventions designed to promote healthy brain aging. Specifically, the present study was motivated by three primary aims. First, we sought to identify distinct phenotypes of Accelerated versus Delayed Brain Aging within a sample of 100 healthy older adults. Brain imaging measures were acquired from a comprehensive battery of over 100 neuroimaging markers of brain health, including measures of brain structure (i.e., volumetrics and white matter tracts), functional brain connectivity, and brain metabolites, as measured by MRS. Second, using a well-validated neuropsychological test battery, we compared performance on measures of intelligence, executive function, and memory in the Accelerated versus Delayed Brain Aging phenotypes. Finally, we investigated whether the observed phenotypes captured distinct nutrient biomarker profiles, with a focus on nutrients that are known to have favorable effects on cognitive function and brain health from the Mediterranean Diet (i.e., fatty acids, antioxidants, and vitamins).

We predicted that phenotypes of Accelerated versus Delayed Brain Aging would emerge, given well-established individual differences in brain aging trajectories. We also predicted that these distinct phenotypes would embody differences in cognitive function manifested by the observed differences in brain aging. Finally, our predictions about the role of nutrition in healthy brain aging were guided by findings to suggest that specific nutrients may benefit brain health, including poly- and mono-unsaturated fatty acids, vitamins, antioxidants, and carotenoids. Thus, by combining advances in Nutritional Cognitive Neuroscience—nutrient biomarkers of diet, multimodal brain imaging, and statistical modeling of brain aging—this interdisciplinary study aimed to identify nutrient profiles associated with Accelerated versus Delayed Brain Aging and to establish nutritional targets for future interventions designed to promote brain health.

## Results

### Brain health phenotypes

A total of 139 variables of brain health were collected in the study, including measures of structure, function, and metabolism (Table 1). Brain volumes were measured separately for the left and right hemispheres for the Structural Regions listed in Table 1, for a total of 68 regions. Total white matter integrity was measured for each of the Structural Regions in Table 1, for a total of 34 tracts. Functional connectivity was assessed using four graph theory metrics on each of 8 brain networks, for a total of 32 measures. Finally, three metabolite concentrations were measured within each brain region.

**Table 1:** Summary of MRI measures used to derive brain health phenotypes

Brain structure^a^		Functional connectivity^b^	
banks superior temporal	para hippocampal	Graph Theory Metrics	Brain Network
caudal anterior cingulate	pars opercularis	Global Efficiency Local Efficiency Small World Propensity Strength	default mode
caudal middle frontal	pars orbitalis		dorsal attention
Cuneus	pars triangularis		frontoparietal
Entorhinal	pericalcarine		limbic
frontal pole	postcentral		motor
Fusiform	posterior cingulate		ventral attention
inferior parietal	precentral		visual
inferior temporal	precuneus		whole brain
Insula	rostral anterior cingulate		
isthmus cingulate	rostral middle frontal	**Brain metabolism**^**c**^	
lateral occipital	superior frontal	Metabolite	Region
lateral orbitofrontal	superior parietal	Choline	Anterior and Posterior Cingulate Cortex
Lingual	superior temporal	Creatine	
medial orbitofrontal	supramarginal	NAA	
middle temporal	temporal pole		
para central	transverse temporal		

^a^The structural regions were measured for both volumetrics (right and left hemispheres measured separately) and white matter.

^b^Functional connectivity included eight brain networks measured by four separate graph theory metrics.

^c^The concentrations of the three brain metabolites were measured in the same region of the brain. *NAA* is N-acetyl aspartate.

Hierarchical clustering of all 139 brain measures collected in the study revealed two distinct phenotypes of brain health: Accelerated and Delayed Aging. Hierarchical clustering uses a similarity metric that identifies individuals who are most like one another across all brain measures. The two phenotypes identified by hierarchical clustering indicate that, across all study participants, one group is similar with less optimal brain health while the other group is similar with more optimal brain health. The dendrogram is presented in Supplementary Fig. 1. The average value for the two phenotypes for each brain imaging domain from Table 1—structure, metabolism, and functional connectivity—is listed in Table 2. Relative to individuals with Accelerated Aging, those with Delayed Aging have larger brain volumes (0.48 vs 0.43), increased white matter integrity (0.61 vs 0.49), increased concentrations of brain metabolites (0.444 vs 0.441), increased functional connectivity for the whole brain (0.51 vs 0.45) and small world propensity (0.52 vs 0.43). Network functional connectivity was greater for individuals with Accelerated Aging relative to those with Delayed Aging for strength (0.57 vs 0.45), local efficiency (0.52 vs 0.47) and global efficiency (0.55 vs 0.43). Supplementary Figs. 2, 3 and 4 present heatmaps of the values for each participant for each network or region of all brain measurements presented in Table 1.

**Table 2:** Average brain measurement values for the Accelerated and Delayed Aging phenotypes of brain health

Domain	Measure	Accelerated Aging	Delayed Aging
Brain Structure	Volumetric Regions	0.43	0.48
	Diffusion Tensor Imaging Tracts	0.49	0.61
Brain Metabolism	Magnetic Resonance Spectroscopy	0.441	0.443
Functional Connectivity	Whole Brain	0.45	0.51
	Small World Propensity (network)	0.43	0.52
	Strength (network)	0.57	0.45
	Local efficiency (network)	0.52	0.47
	Global efficiency (network)	0.55	0.43

Values were averaged across all variables listed in Table 1. Before averaging, values for each variable were scaled between 0 and 1.

### Brain age and brain health phenotypes

**Fig. 1:**
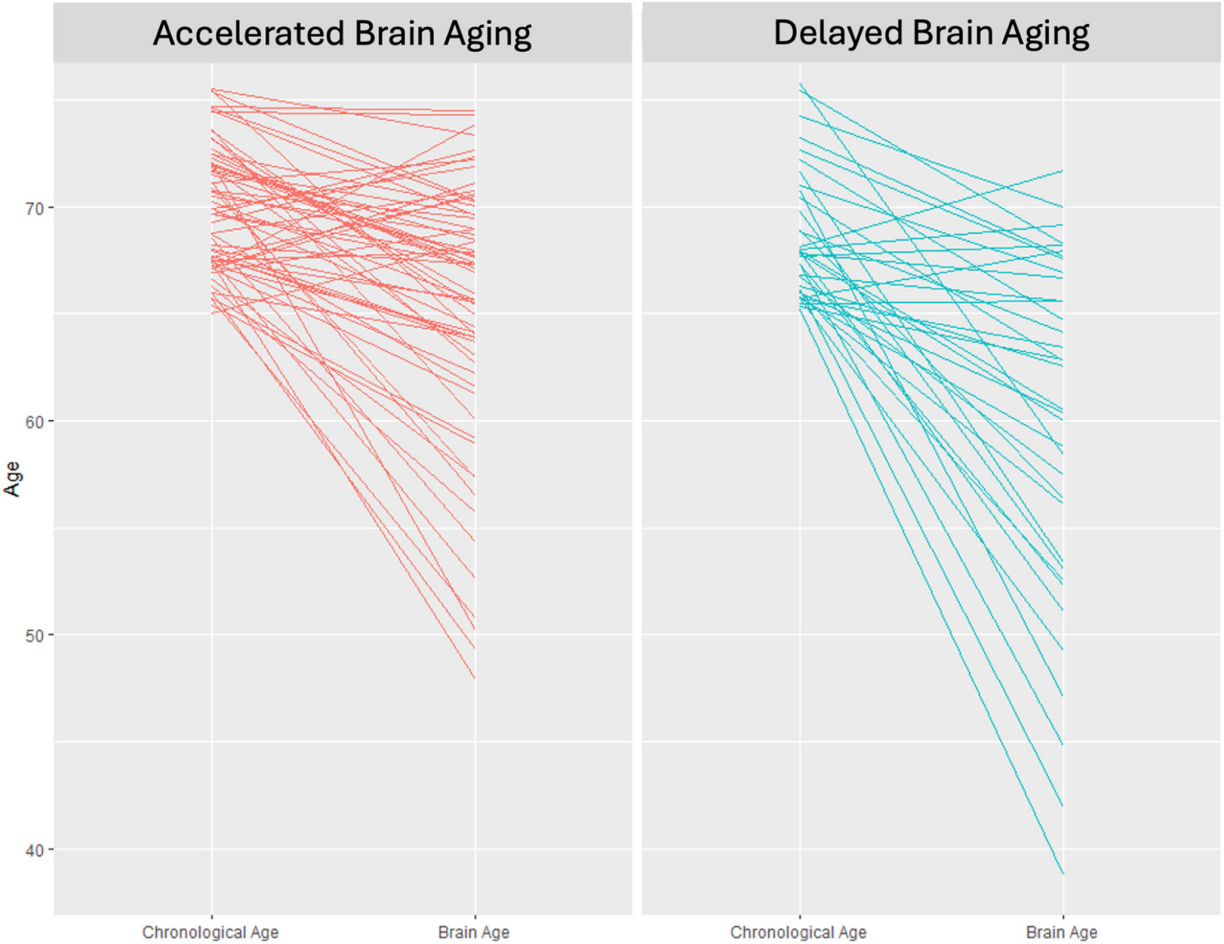
Chronological versus brain age. Chronological age (left side of each panel) versus brain age (right side of each panel) for the accelerated (left panel) and delayed (right panel) brain aging phenotypes.

Brain Age (BA) was estimated for both brain health phenotypes. Individuals in the Accelerated Aging phenotype have an average BA of 65.1 whereas those in the Delayed Aging have an average BA of 59.7. This difference of 5.4 BA years is significant (*t* statistic = 2.66, *p* value = 0.010). Chronological Age (CA) and BA are presented in Fig. 1 for both brain phenotypes. Many individuals in the Accelerated Aging phenotype have BAs closer to their CAs whereas many individuals in the Delayed Aging phenotype have BAs less than their CAs. Correlations between BA were computed with multiple brain modalities. BA was negatively associated with brain volumes (*r* = −0.19, *p* value = 0.058), white matter tracts (*r* = −0.37, *p* value = 0.00014), brain metabolites (*r* = −0.25, *p* value = 0.017) and functional connectivity measures (*r* = −0.19, *p* value = 0.063). These robust negative correlations suggest that a young BA is associated with the Delayed Brain Aging phenotype, indicating multiple brain modalities can predict BA specifically and brain health generally.

### Brain health and cognition

**Fig. 2:**
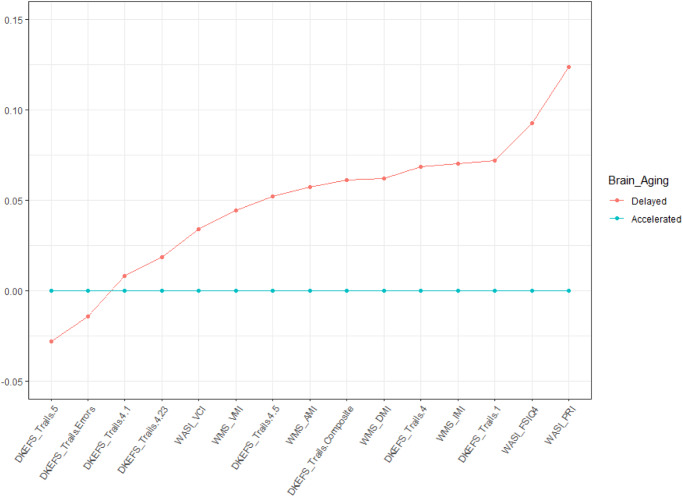
Cognitive performance of the Delayed and Accelerated Brain Aging groups for 15 assessments of intelligence (WASI), executive function (DKEFS), and memory (WMS). The scores for Accelerated Brain Aging were set to a baseline of 0 for each cognitive measure and the Delayed Brain Aging group is expressed as a difference relative to that baseline, with values further from the baseline reflecting a larger difference. All cognitive scores were scaled between 0 and 1.

The study results established robust differences in brain structure, function, and metabolism between the two phenotypes of brain health. Furthermore, these phenotypes were inversely correlated with BA, where the individuals with Accelerated Aging have an Old BA and those with Delayed Aging have a Young BA. We next examined how differences in Delayed and Accelerated Brain Aging phenotypes map onto cognition. We assessed measures of intelligence (WASI), executive function (DKEFS), and memory (WMS). Individuals with Delayed Brain Aging outperformed those with Accelerated Brain Aging for all cognitive tests (see Fig. 2). Two scores for the Delayed Brain Aging Phenotype, reaction time (DKEFS Trails 5) and response errors (DKEFS Trails Errors), are negative and smaller than the Accelerated Brain Aging Phenotype, reflecting better cognitive performance for the Delayed Brain Aging Phenotype. A *t* test comparing the means between the Delayed and Accelerated Brain Aging groups is significant (p-value < 0.05) for tests of general and fluid intelligence (WASI_FSIQ4 and WASI_PRI), executive function (DKEFS_Trails.1) and memory (WMS_IMI). Supplementary Table 1 includes the scaled scores for all 15 cognitive tests.

### Nutrient profile of healthy brain aging

Having established two distinct phenotypes of brain aging derived from measures of brain structure, function, and metabolism, and based on a comprehensive cognitive battery, we lastly determined the nutritional profile of the Delayed Brain Aging phenotype (illustrated in Table 3). We examined the nutritional status of study participants using blood-based biomarkers. The nutrient biomarker profile of individuals in the Delayed Brain Aging phenotype was characterized by greater concentrations of 13 key nutrients compared to the Accelerated Aging phenotype, as determined by a t-test comparing the group means. Notably, the observed nutrient profile encompassed several nutrient categories important for brain health, including monounsaturated fatty acids (C18:1n-7 and C20:1n-9), ω-3 polyunsaturated fatty acids (C18:3n-3 and C20:5n-3, commonly known as ALA and EPA, respectively), ω-6 polyunsaturated fatty acids (C22:2n-6 and C20:2n-6), one long-chain saturated fatty acid (C24:0), the carotenoids lutein and zeaxanthin, and vitamin E and choline (see Supplementary Table 2). Nutrients that did not differ between the Accelerated versus Delayed Brain Aging phenotypes include Vitamins A, B2, B6, B12, D and E, carotenoids lycopene and carotene, short and medium chain saturated fatty acids (i.e., C10:0 to C22:0), and some PUFAs (e.g., C18:4n-3 and C22:4n-6). The coefficient of variability and intraclass correlation coefficient for the blood biomarkers in Table 3 are presented in Supplementary Table 3.

**Table 3:** Nutrient profile of the Delayed Brain Aging phenotype

Nutrient Category	Nutrient	Name
Fatty Acids	C18:1n-7	Vaccenic Acid
	C20:1n-9	Gondoic Acid
	C18:3n-3	Alpha Linolenic Acid (ALA)
	C20:5n-3	Eicosapentaenoic Acid (EPA)
	C22:2n-6	Docosadienoic Acid
	C20:2n-6	Eicosadienoic Acid
	C24:0	Lignoceric Acid
Antioxidants and Carotenoids	cis-lutein	Lutein
	trans-lutein	Lutein
	zeaxanthin	Zeaxanthin
Vitamins and Vitamin-Like Compounds	α-tocopherol	Vitamin E
	γ-tocopherol	Vitamin E
	Choline	Choline

Additionally, to rule out the possibility that other covariates contributed to brain aging differences between the phenotypes, we investigated multiple measures of demographics, anthropometrics, and physical fitness collected in the study. None of these variables differed between the two phenotypes using a t-test of the means, suggesting these factors do not explain the observed differences in brain aging (see Supplementary Table 4). Finally, following standard conventions, we controlled for BMI, sex, income, and education by including these factors as covariates in the analysis.

## Discussion

Nutritional Cognitive Neuroscience aims to identify specific foods and nutrients that promote healthy brain aging. Central to this effort is the discovery of nutrient profiles that can be targeted in nutritional interventions designed to promote brain health with respect to multimodal neuroimaging measures of brain structure, function, and metabolism. The present study advances four primary conclusions pursuant to this goal.

First, we provide evidence for a multimodal characterization of healthy brain aging, classified according to neuroimaging measures associated with Delayed or Accelerated Brain Aging. Specifically, relative to Accelerated Brain Aging, older adults with Delayed Brain Aging exhibited: (1) larger brain volumes, (2) better structural DTI integrity across 34 brain regions, (3) better functional connectivity for whole brain and network-level measures of local and global efficiency, and for small world propensity, and (4) greater concentrations of the brain metabolites choline, creatine, and NAA. These findings are notable given that prior research has focused primarily on a single brain imaging modality, limiting the nature and scope of conclusions drawn about the role of diet and nutrition in healthy brain aging.

Second, Delayed Brain Aging was inversely correlated with neuroimaging biomarkers of BA, demonstrating that a younger BA is associated with favorable brain health outcomes with respect to measures of brain structure, function, and metabolism^35^.

Third, scores on cognitive assessments of intelligence, executive function, and memory were higher for older adults with Delayed Brain Aging compared to those with Accelerated Brain Aging. These results are consistently supported by findings from the cognitive neuroscience of aging, namely that older adults with larger brain volumes, white matter tracts with greater integrity, and more efficient functional connectivity also demonstrate better cognitive performance^26–28^.

Fourth, the discovered nutrient profile for healthy brain aging was not derived from dietary questionnaires, as is typically employed in nutritional epidemiology, but from nutrient biomarkers which accurately reflect the concentrations of nutrients from the diet^36,37^. The observed nutrient profile is both broad, including fatty acids, carotenoids, and vitamins, and specific, identifying the amount and type of specific nutrients in each category.

Finally, the current study identified a nutrient profile related to healthy brain aging and a clinically relevant neuroimaging biomarker of BA. Although nutrition represents an established risk factor for age-related neurological disease^34^, the potential benefits of nutrition for promoting brain health are less well understood. Thus, the nutrient pattern identified by the present study motivates the design of neuroscience-guided dietary interventions to promote healthy brain aging. We now review the primary elements of the observed nutritional pattern and the mechanisms of action that have been proposed in the nutritional sciences to explain their benefits on cognitive and brain health.

### Fatty acids

In our study, the fatty acid nutritional profile of individuals in the Delayed Brain Aging phenotype includes increased concentrations of EPA, ALA, docosadienoic acid, and eicosadienoic acid, all of which are known to reduce inflammation. Inflammation makes the vascular blood–brain barrier more permeable to cytokines, and chemokines, which interfere with neuronal and glial well-being and interrupt brain homeostasis^38–40^. Accumulating evidence has linked increased inflammation to decline in brain structure and function, cognitive decline, and increased risk of dementia^41–43^. We review the relationship between inflammation and each of these fatty acids in turn below.

Eicosanoids, signaling molecules responsible for cellular functions regulating inflammation and the central nervous system, derive from competing metabolic pathways that begin with three different 20-carbon fatty acids: arachidonic acid (20:4 ω-6, AA), eicosapentaenoic acid (20:5 ω-3, EPA), and di-homo γ linolenic acid (20:3 ω-6, DGLA)^44^. The ω-6 AA pathway promotes inflammation whereas the ω-3 DGLA and EPA pathways are less inflammatory, biologically inert, or even anti-inflammatory. Moreover, the three pathways compete for the same enzymes, rate-limiting molecules, transport, and acylation pathways. Hence, the greater the presence of anti-inflammatory generating ω-3 EPA in the diet, as is observed in our nutrient profile of Delayed Brain Aging, the fewer inflammatory eicosanoids will be generated by the AA pathway.

Another nutrient in the Delayed Brain Aging phenotype related to EPA and beneficial inflammation is α-linolenic acid or ALA. ALA is one of two essential fatty acids. EPA is obtained through diet or endogenously produced, via a metabolic pathway beginning with ALA. ALA converts to EPA with 10–20% efficiency^45^. Thus, greater concentrations of ALA in the diet, as is the case for individuals with healthy brain aging, can theoretically yield more of the beneficial EPA to compete with the inflammatory AA cascade. Excellent dietary sources of EPA and ALA include fish and shellfish, flaxseed, hemp seed, olive oil, soya oil, canola oil, chia seeds, pumpkin seeds, sunflower seeds, leafy vegetables, and walnuts.

The DGLA pathway is another beneficial competitor to the AA cascade, resulting in less severe inflammation or even anti-inflammatory metabolites. Two other nutrients in the profile of Delayed Brain Aging, eicosadienoic and docosadienoic acids, are closely related to the DGLA pathway. Eicosadienoic acid is the direct precursor of DGLA while docosadienoic acid is the immediate elongation product of DGLA. These two fatty acids have antioxidant abilities and anti-inflammatory properties meeting or exceeding those of DHA^46^. They also exhibit inhibitory activity against inflammation-causing enzymes by exerting similar physiological effects as over the counter non-steroidal anti-inflammatory drugs (e.g., ibuprofen) that block the COX-I and COX-II enzymes responsible for inflammation and pain^47^.

Saturated fatty acids are traditionally viewed as unhealthy^48^. However recent research suggests some long-chain fatty acids, with more than 20 carbon atoms, may confer health benefits, with evidence suggesting that they are associated with lower risk of coronary heart disease and type 2 diabetes, and may promote healthy aging^49^. Our nutrient profile of Delayed Brain Aging includes one very long chain saturated fatty acid, lignoceric acid (C24.0). Peanuts, macadamia nuts, and certain seed oils are excellent sources of lignoceric acid^49^. A recent study demonstrated that higher concentrations of long-chain fatty acids in plasma in mid-life resulted in reduced cognitive decline in a test of verbal fluency 20 years later^50^. Another study examined the concentration of lignoceric acid in brain tissue and discovered that females without cognitive impairment exhibited a larger concentration of C24.0 compared to females who developed Alzheimer’s disease. These findings provide a rationale for suggesting that long-chain saturated fatty acids, and lignoceric acid specifically, are important biomarkers of cognitive and brain health.

The final two fatty acids discovered in our nutrient profile of Delayed Brain Aging, vaccenic and gondoic acids, are both mono-unsaturated fatty acids, or MUFAs. MUFAs, which are common in olive oil and the Mediterranean Diet, are known to support brain and cognitive health^51^. Both vaccenic and gondoic acid have robust antioxidant activities^47^. Vaccenic acid is the primary type of fat from dairy products, such as milk, butter, and yogurt. Increasing consumption of dairy products increases the concentration of vaccenic acid in plasma^52^. The importance of vaccenic acid for brain health may lie in its metabolic conversion to conjugated linoleic acid, or CLA^53^. CLA is incorporated and metabolized into brain tissue, which further extends its anti-neuroinflammatory properties^54^. Nervonic acid is the predominant fatty acid in the white matter tissue of humans and one of its metabolic precursors is gondoic acid^55^. Based on these findings, it is possible that increased dietary concentrations of vaccenic and gondoic acids may enhance white matter brain integrity, although future research in Nutritional Cognitive Neuroscience is needed to clarify the precise role of these MUFAs in brain and cognitive health.

### Carotenoids

Three different carotenoids, phytopigments that give many fruits and vegetables their characteristic color, figure prominently in the nutritional profile of Delayed Brain Aging. Carotenoid-rich foods include spinach, kale, corn, bell peppers (red, green, or yellow), tomatoes, watermelon, grapefruit, cantaloupe, broccoli, and carrots. Carotenoids have known benefits to cognitive and brain health, as demonstrated by studies that examine their effects on brain structure, brain network function, and memory^7,56–58^. Carotenoids accumulate in the retina of the eye and in the brain, and greater consumption of carotenoids increases their concentration in these tissues^59^. Carotenoids are known to benefit the brain because of their antioxidant properties. The brain is particularly vulnerable to oxidative stress due to its high lipid concentrations and high energy requirements^60^.

### Vitamins

Vitamin E and choline were identified as important nutrients that promote cognitive and brain health in the Delayed Brain Aging phenotype. Multiple studies, including RCTs, have shown that high concentrations of Vitamin E in plasma are associated with better cognitive performance in healthy populations, aging populations, and Alzheimer disease patients^56,61^. Vitamin E’s efficacy in mitigating cognitive decline is likely through its antioxidant properties and its ability to aid in the transporting of fatty acids^62^. A recent RCT demonstrated that supplementation of Vitamin E, along with ω-3 fatty acids and carotenoids, improves performance on tests of working memory^57^. These findings are consistent with the results of the present study, which observed higher scores on tests of intelligence and memory within the Delayed Brain Aging phenotype. Notably, intelligence and memory are supported by multiple cortical regions (e.g., prefrontal, cingulate, and parietal cortices) and networks (e.g., frontoparietal network, the default mode network, and the salience network)^63^. Within the Delayed Brain Aging phenotype, we observed that these regions and networks demonstrated superior performance compared to the Accelerated Brain Aging phenotype based on measures of cortical volume and functional brain network efficiency, respectively. Excellent dietary sources of Vitamin E include nuts, seeds, and vegetable oils while significant amounts also come from green leafy vegetables and fortified cereals.

Choline, an essential B-vitamin-like nutrient, is also in the nutrient profile of Delayed Brain Aging. Choline plays at least two critical functions for cognitive and brain health: it is a necessary precursor for phosphatidylcholine, the predominant lipid in cell membranes and it is required for the synthesis and release of acetylcholine, a critical neurotransmitter^64^. Furthermore, brain white matter tracts and brain volume, which are enhanced in the Delayed Brain Aging phenotype, critically depend on choline for their cellular structure and integrity. Choline benefits both executive function and memory^15,64^. Excellent dietary sources of choline include animal-based proteins such as meat, poultry, fish, and eggs, while cruciferous vegetables and certain beans are also rich in choline.

Overall, there is strong evidence to support the nutrient profile underlying the Delayed Brain Aging phenotype in promoting cognitive and brain health. Many of the biochemical pathways underlying fatty acid synthesis and metabolism are well-known; but the implications of those competing pathways for cognitive and brain health, which importantly depend on the nutrients available from the diet, are only beginning to be understood. Moreover, future research should examine the differential impact of nutrition on different brain regions and networks, as certain nutrients may be important for different regions of the brain whereas other nutrients are required by the brain globally. Applying methods from Nutritional Cognitive Neuroscience, future RCTs should systematically investigate the effects of specific nutrient profiles on the structural integrity and functional efficiency of specific cortical regions and networks (e.g., combining nutrient biomarker analysis with MRI measures of local and global brain network operations). The current results provide evidence that some metabolic pathways (e.g., the DGLA pathway compared to the AA pathway) may yield more optimal brain and cognitive outcomes. Furthermore, the observed metabolic pathways that are less optimal for cognitive and brain health often result in higher levels of inflammation. Carotenoids and vitamins identified in the current study that benefit cognitive and brain health, such as lutein, choline, and Vitamin E, require regular consumption to have their beneficial effect. Importantly, these nutrients may accumulate preferentially in certain brain regions or networks, motivating an investigation of the selectivity of nutrition for promoting the health and function of specific brain regions and cortical networks.

### Limitations

While some of the nutrients observed in the present study have solid molecular mechanisms to help explain their role in cognitive and brain health, other nutrients are less well understood. Research on long-chain saturated fatty acids and MUFAs (vaccenic acid and gondoic acid) have several preliminary studies suggesting their benefits on cognitive and brain health, but more research is needed to establish the precise mechanisms by which they exert an effect. Another limitation of the present study concerns the cross-sectional study design, sample size, and Caucasian participants. The Delayed Brain Aging phenotype identifies a set of nutrients that longitudinal and randomized controlled trials should target in future studies to determine their effects on cognitive and brain aging. Other statistical tools, such as canonical correlation analysis and structural equation modeling, may also be applied to draw new insights about the associations between brain aging and nutrition. The results of the present study need to be examined in non-Caucasian participants to assess the generality of findings. Moreover, while the current study examined brain health applying measures of brain structure, function, and metabolism, future research should also seek to understand how diet and nutrition effect the trajectory of brain aging within each of these measures. Additionally, the nutrient profile of Delayed Brain Aging identified in the current study does not imply they are the only nutrients that matter for brain health. Clearly, the brain needs many nutrients for healthy functioning, including amino acids, multiple B vitamins, ω-3 and ω-6 polyunsaturated fatty acids, monounsaturated fatty acids, choline, Vitamins C and D, and minerals like iron, zinc, and magnesium^65^. The nutrient profile identified in the current study differentiates Delayed Brain Aging from Accelerated Brain Aging. Finally, while the nutrient profiles discovered here suggest a basis for future testing of dietary interventions for optimal brain health, additional studies are needed to further establish and validate the present findings. It will also be important to build large-scale studies and research consortia to investigate the relationship between alternate measures of dietary intake and nutritional status, examining the reliability and validity of nutrient biomarkers, food frequency questionnaires, and their respective merits and limitations^66,67^.

## Conclusion

The present study identified a specific profile of nutrients that may promote healthy brain aging, motivating further research to establish and validate these findings in the context of a randomized controlled trial. By building upon the observed findings, future research can inform the development of more effective, targeted dietary interventions that apply methods in Nutritional Cognitive Neuroscience. We believe this approach holds promise for the development of dietary strategies to support cognitive function and brain health in the aging population.

## Methods

### Population

This cross-sectional study enrolled 100 healthy elderly adults from the Illinois Brain Aging Study cohort, a sample of community-dwelling Caucasian men and women aged 65–75 years. Participants were neurologically healthy and did not have evidence of cognitive impairment, as determined by a score of lower than 26 on the Mini-Mental State Examination^68^. Participants with mild cognitive impairment, dementia, a psychiatric illness within the last three years, a stroke within the past twelve months, cancer in the last three years, an inability to complete study activities, prior involvement in cognitive training or dietary intervention studies, or contraindications for magnetic resonance imaging (MRI) were excluded. All participants were right-handed with normal, or corrected to normal, vision.

### Standard protocol approval and patient consent

In accordance with the University of Illinois and Carle Foundation Hospital Institutional Review Boards, informed consent was obtained from all participants in this study.

### Nutrient biomarker acquisition and analysis

Fasting plasma was collected from each participant between 7:00 AM and 12:00 noon Central Time. Nutrient biomarkers were assayed, comprising three general classes of nutrients: fatty acids, carotenoids, and vitamins. Ethylenediaminetetraacetic acid (EDTA) plasma carotenoids and tocopherols were analyzed by high-performance liquid chromatography with a photodiode array detector (HPLC-PDA) using UV detection^69^. Plasma lipids were measured with gas chromatography using flame ionization and peaks of interest were identified by comparison to authentic fatty acid standards^70^. Vitamins were measured by a chemiluminescent immunometric assay or after extraction by radioimmunoassay^71,72^.

### MRI data acquisition and processing

All data were collected on a Siemens Magnetom 3T Trio scanner using a 32-channel head coil in the MRI Laboratory of the Beckman Institute Biomedical Imaging Center at the University of Illinois.

### MRI data acquisition

A high-resolution multi-echo T1-weighted magnetization prepared gradient-echo structural image was acquired for each participant (0.9 mm isotropic, TR: 1900 ms, TI: 900 ms, TE = 2.32 ms, with GRAPPA and an acceleration factor of 2). The functional neuroimaging data were acquired using an accelerated gradient-echo echoplanar imaging sequence sensitive to blood oxygenation level dependent (BOLD) contrast (2.5 × 2.5 × 3.0 mm voxel size, 38 slices with 10% slice gap, TR = 2000 ms, TE = 25 ms, FOV = 230 mm, 90° flip angle, 7 min acquisition time). During the resting-state fMRI scan, participants were shown a white crosshair on a black background viewed on an LCD monitor through a head coil-mounted mirror. Participants were instructed to lie still, focus on the visually presented crosshair, and to keep their eyes open^73^.

### MRI data preprocessing

All MRI data processing was performed using FSL tools available in Functional Magnetic Resonance Imaging of the Brain (FMRIB) Software Library version 5.0. The high-resolution T1 Magnetization-Prepared Rapid Gradient-Echo (MPRAGE) was extracted using the Brain Extraction Tool^74^. FMRIB’s Automated Segmentation Tool^75^ was applied to delineate gray matter, white matter, and cerebral spinal fluid (CSF) voxels. The resting-state fMRI data were pre-processed using the FSL FMRI Preprocessing and Model-Based Analysis (FEAT) analysis tool^76,77^. Pre-processing entailed: slice timing correction, motion correction, spatial smoothing (3 mm full width at half maximum kernel), nuisance signal regression (described below), standard fMRI temporal bandpass filtering (0.009–0.1 Hz, linear registration of functional images to structural images, and non-linear registration of structural images to the MNI152 brain template (2 mm isotropic voxel resolution).

Nuisance variables were modeled via General Linear Modeling (GLM) analyses to remove spurious correlations, noise introduced by head motion, and variables of no interest. These included head motion correction parameters (using the extended 12 motion parameters estimated in FEAT preprocessing), modeling of individual volume motion outliers estimated using DVARS (outliers flagged using the boxplot cutoff 1.5 × interquartile range^76^), and averaging of mean white matter and cerebrospinal fluid signals across all voxels identified from the segmentation of the high resolution MPRAGE. The fully preprocessed resting-state fMRI data comprised the residual obtained from fitting these nuisance variables in the GLM framework. The residuals were transformed into normalized MNI152 space and re-sampled to 4 mm isotropic voxels to reduce computational complexity in post data processing for network analysis.

### Brain volumetrics

Cortical reconstruction was performed with the Freesurfer image analysis software^78^. For this analysis, all the cortical gray matter volumes provided by the Freesurfer parcellation were examined. This included 68 regions throughout the frontal, parietal, temporal, and occipital lobes. Volumetric measures were adjusted for intracranial volume and sex using a regression model. The adjusted values were then used in further statistical analyses.

Volumetric analysis was performed on data from a 3D high-resolution (0.9 mm isotropic) T1-weighted scan using MPRAGE acquisition. Cortical reconstruction was performed with the Freesurfer image analysis suite, which is documented and freely available for download online (http://surfer.nmr.mgh. harvard.edu/). All cortical reconstructions were manually checked for accuracy, as recommended by the software developers. The volumetric analyses focused on gray matter volume in the temporal cortex provided by Freesurfer parcellation. Regions of interest included the superior temporal cortex, middle temporal cortex, inferior temporal cortex, banks of the superior temporal sulcus, fusiform cortex, transverse temporal cortex, entorhinal cortex, temporal pole, and parahippocampal cortex.

### Diffusion tractography imaging (DTI)

Whole brain diffusion tensor imaging was acquired with the following parameters: FOV = 240 × 240 mm; 72 slices, slice thickness = 2 mm; TE = 98 ms; TR = 10,000 ms; in-plane resolution = 1.875 × 1.875 mm; diffusion encoding directions = 30; *b* = 0 s/mm^2^ and 1000 s/mm^2^. Data were processed using the University of Oxford’s Center for FMRIB Software Library (FSL) release 5.0^79^ diffusion toolbox^80,81^. Eddy current correction was accomplished using the eddy correction tool and a diffusion tensor model was fit in each voxel using the DTIFIT tool, which generates fractional anisotropy (FA) values in every voxel. FA images were further processed using the FSL tract-based spatial statistics^82^ toolbox, which projects each subjects’ FA data onto a mean white matter skeleton, representing the white-matter tracts common to all subjects. Mean FA within the white matter skeleton for specific regions of interest were calculated for each subject using the JHU ICBM DTI-81 atlas^83^.

### Graph theory metrics of brain efficiency

The efficiency of brain network function was examined by investigating the small-world organization^84^ of seven well-established intrinsic connectivity networks of the brain^85^. A small-word organization represents the optimal balance of local and global network efficiency, providing a parsimonious neural architecture that supports high local clustering (local efficiency) and short average path length (global efficiency). The procedure for computing small-world propensity is presented below.

First, the mean fMRI BOLD time series was extracted from subjects’ gray matter voxels using the Craddock parcellated brain atlas as a mask^86^. This parcellation of 200 regions provided whole-brain coverage and sufficiently high spatial resolution for conducting network analysis. A subject-wise functional connectivity matrix reflecting pairwise Pearson correlations between the mean BOLD time series signals obtained from nodes defined by the Craddock atlas was then computed and Fisher’s Z-transformed to achieve normality. These were standardized to Z-scores through multiplication with their standard deviation approximated as *σ* = 1/√(*n* − 3), where *n* is the number of time points corresponding to the BOLD signal^87^. A Bonferroni-corrected statistical Z-threshold was applied to identify significant positive correlations (*p* < 0.05) within each subject’s whole-brain functional connectivity matrix derived from Craddock’s 800 parcellation atlas^88,89^. The thresholded Z-scores were rescaled to represent connection weights ranging from 0 to 1. Based on these positive connection weights, weighted connectivity matrices representing functional connectivity between nodes representative of seven intrinsic connectivity networks were obtained for each subject. The seven intrinsic connectivity network maps—visual, somatosensory, limbic, default mode, dorsal attention, ventral attention and frontoparietal—are at https://surfer.nmr.mgh.harvard.edu/fswiki/CorticalParcellation_Yeo2011.

We then examined small-world propensity within the rescaled connectivity matrices derived for each of the seven intrinsic connectivity networks. Small world propensity *Φ* is calculated as the fractional deviation between a network’s clustering coefficient, *C*_*obs*_, and characteristic path length, *L*_*obs*_, from both lattice (*C*_*latt*_, *L*_*latt*_) and random (*C*_*rand*_, *L*_*rand*_) networks constructed with the same number of nodes and the same degree distribution:

1 $$\phi =1-\sqrt{\frac{{\Delta }_{C}^{2}+{\Delta }_{L}^{2}}{2}}$$

where,

2 $${\Delta }_{C}=\frac{{C}_{{latt}}-{C}_{{obs}}}{{C}_{{latt}}-{C}_{{rand}}}$$

and

3 $${\Delta }_{L}=\frac{{L}_{{obs}}-{L}_{{rand}}}{{L}_{{latt}}-{L}_{{rand}}}$$

The ratios Δ_*C*_ and Δ_*L*_ represent the fractional deviation of the metric (*C*_*obs*_ or *L*_*obs*_) from its respective null model (a lattice or random network).

### Magnetic resonance spectroscopy (MRS)

Metabolite concentration from MRS was determined using the procedure in ref. ^90^. The anatomical scan was used to position a single voxel spectroscopy (SVS) scan in the parietal cortex extending into posterior cingulate cortex (voxel size: (20 mm), TR: 3000 ms, TE: 30 ms, 40 averages, BW: 2000 Hz, vector size: 1024). The voxel straddled the midline, including equal portions of the right and left hemispheres. Weak water suppression was employed, and six regional saturation bands were placed around the voxel to reduce contamination from subcutaneous fat. An additional scan was performed without water suppression to aid with quantification. Immediately following the spectroscopy acquisition, a T2-weighted overlay scan was performed with the same center location and orientation as the spectroscopy scan (TR = 5000 ms, TE = 84 ms, slice thickness 2 mm with 0.5 mm of spacing, FOV: 240 × 240 mm, 128 × 128 matrix size, GRAPPA acceleration factor: 2, 35 slices).

Metabolite quantitation was performed using tissue water as a reference. Water-scaled spectra were analyzed using LCModel software (Version 6.3-1H). No correction was performed to account for relaxation of metabolite signal. Because NAA and NAAG are difficult to differentiate, here we analyze the combined concentration of NAA + NAAG, labeled herein as NAAt with a peak appearing at 2.02 ppm.

Accurate water scaling requires corrections for the tissue composition of the voxel. Using the high-resolution structural scan, we calculated the volume fractions of gray matter (GM), white matter (WM), and CSF within each voxel using Matlab scripts (MathWorks, Natick, MA) that called functions from SPM8 (Wellcome Trust Centre for Neuroimaging). First, we segmented the MPRAGE using SPM8 to obtain tissue probability maps of GM, WM and CSF. We then created a mask in the space of the T2 overlay corresponding to the location of the spectroscopy scan. This mask has the same center and orientation as the T2 overlay but higher resolution (0.5 × 0.5 × 0.5 mm). We then registered the MPRAGE to the T2 overlay. The rotations and translations required for the registration were then applied to the tissue probability masks. We resliced the tissue probability maps into the space of the mask, and used the mask to calculate the volume fractions of GM, WM, and CSF within the volume of the spectroscopy voxel. These tissue fractions were later used to statistically correct NAAt for tissue volume-fraction dependencies.

### Brain age (BA)

BA was derived using Brain-Age Regression Analysis and Computation Utility Software, or BARACUS, using T1-weighted structural images^33,91^.

### Cognitive assessments

Neuropsychological tests investigating multiple facets of memory, executive function, and intelligence were administered. Our battery included the Wechsler Adult Intelligence Scale (WAIS^92^), the trail-making test from the Delis-Kaplan Executive Function System (DKEFS^93^), and the Wechsler Memory Systems (WMS^94^).

### Memory

Memory was measured by the Wechsler Memory Scale—Fourth Edition (WMS-IV) Older Adult Battery^94^. This assessment measured memory by way of four indices: Auditory Memory Index, Visual Memory Index, Immediate Memory Index, and Delayed Memory Index. The Auditory Memory Index indicates a participant’s ability to remember orally presented information. The Visual Memory Index indicates a participant’s ability to remember visually presented information. The Immediate Memory Index indicates a participant’s ability to recall visually and orally presented information immediately after it is presented. The Delayed Memory Index indicates a participant’s ability to recall and recognize visually- and orally-presented information after a 20 to 30-min delay. Participants’ raw scores on each subtest were converted to normalized scaled scores and subsequently combined into indices. Z-scores for each index were calculated and then averaged to create a composite memory score^95^.

### Executive function

Executive functions were measured by the Delis–Kaplan Executive Function System (D–KEFS) Trail Making Test^93^. This assessment yields a measure of the executive functions that can be isolated from underlying skills, including visual scanning, number sequencing, letter sequencing, and motor speed. In this task, participants alternate between multiple task goals (either number or letter sequencing), which elicits a specific component of the executive functions known as cognitive flexibility. The reported results from the D-KEFS Trail Making Test assess cognitive flexibility while controlling for number and letter sequencing trials and therefore provide a measure of cognitive flexibility that is not confounded by underlying cognitive abilities (i.e., number and letter sequencing) required by the task.

### Intelligence

General intelligence was measured by the Wechsler Abbreviated Scale of Intelligence– second edition (WASI-II^92^). Per the scoring guidelines, this assessment measured general intelligence by way of an estimated intelligence quotient score, derived from fluid and crystallized test scores which, in turn, were derived from four subtests: a block design subtest, a matrix reasoning subtest, a vocabulary subtest, and a similarities subtest. In the block design subtest, participants were asked to reproduce pictured designs using specifically designed blocks as quickly and accurately as possible. In the matrix reasoning subtest, participants were asked to complete a matrix or serial reasoning problem by selecting the missing section from five response items. In the vocabulary subtest, participants were asked to verbally define vocabulary words (i.e., What does lamp mean?) that became progressively more challenging. In the similarities subtest, participants were asked to relate pairs of concepts (i.e., How are a cow and bear alike?) that became progressively more challenging. Per scoring guidelines, subjects’ raw scores were converted to standardized scores and combined into an estimated intelligence quotient score, which provided a measure of general intelligence.

### Demographics, anthropometrics, and fitness

Demographics, including sex, education, and income, were collected via questionnaire responses provided by the participants. Anthropometric measures of weight, height, hip, and waist circumference were assessed by the study coordinator at the first study visit. Several measures of physical fitness were also collected or determined. Body Mass Index (BMI) was calculated from measured height and weight. Resting Heart Rate (RHR) was measured by the study coordinator during the participant’s first visit to the lab. Physical activity and the metabolic equivalent of VO_2_ were assessed via a published questionnaire and required BMI and RHR as inputs^96^.

### Analysis overview

First, individual differences in brain health were determined from the multimodal neuroimaging features using hierarchical clustering. Individuals with similar brain health were grouped together. Second, brain aging trajectories were computed for everyone and for each brain health group. Third, we examined cognitive aging differences within each brain health group. Fourth, a nutrient profile for the healthy brain and cognitive aging group was determined. Finally, lifestyle factors other than diet were examined to determine their role in brain and cognitive aging.

### Statistical analyses

All statistical analyses were conducted in R Studio Version 2022.2.3.492^97^ using the R statistical computing software environment Version 4.0.3^98^. Covariates with the weakest inter-correlations associated with age-related cognitive decline (age, gender, education, income, and Body Mass Index) were included as predictors in a separate regression model for each outcome measure of nutrition, brain, and cognition. Residualization was performed using the Frisch-Waugh-Lowell theorem. The net result of residualization is to remove the potential effects of covariates on the outcome. The residuals from each model were normally transformed using Tukey’s Ladder of Powers^99^ and then [0,1] scaled. This process also had the effect of transforming outlier values to a normal range and resulting in a range of 0 to 1 for all variables. Hierarchical clustering, using the complete linkage method, was used to cluster individuals according to similarity of their brain features and it is not sensitive to multicollinearity. *T* tests of the mean were used to assess the magnitude and significance of differences between phenotypes.

### Reporting summary

Further information on research design is available in the Nature Research Reporting Summary linked to this article.

### Supplementary information

Supplemental Information

Reporting Summary

## Data Availability

The individual de-identified participant data can be made available upon request.

## Code availability

Codes used for this study are available upon reasonable request from the authors.
