# Supplementary material for: Investigating nutrient biomarkers of healthy brain aging: a multimodal brain imaging study
Source: NPJ Aging. 2024 May 21;10(1):27. doi: 10.1038/s41514-024-00150-8 (PMC11109270; doi:10.1038/s41514-024-00150-8)
Supplement: Supplementary file 1 — Supplemental Information [file 41514_2024_150_MOESM1_ESM.pdf]

Supplementary Information

Supplementary Figure 1

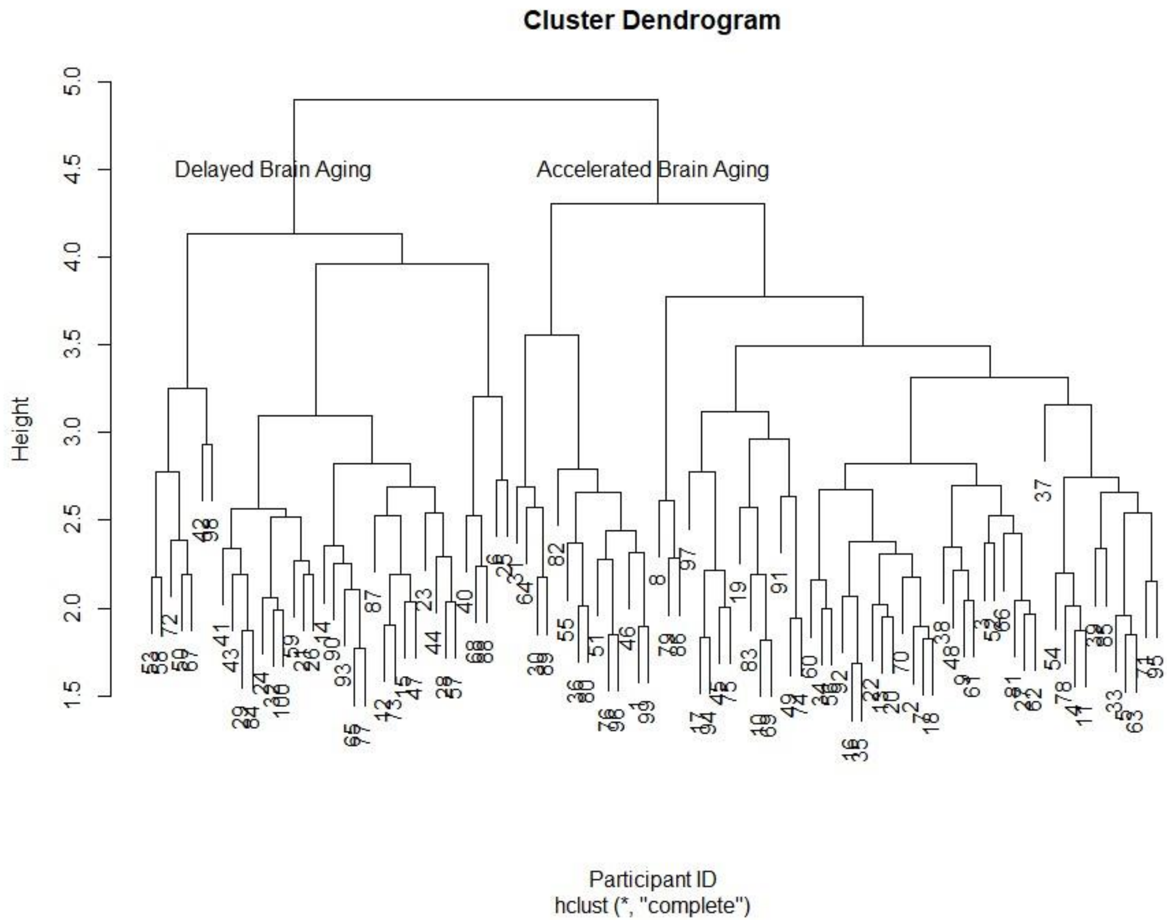

Delayed Brain Aging represents the participants on the left side of the dendrogram and Accelerated Brain Aging represents the participants on the right side of the dendrogram.

Supplementary Figure 2

Integrity of 34 diffusion tensor imaging white matter tracts. A 0 (purple) is weakest integrity and 1 (yellow) is strongest integrity. Each row represents one white matter tract. Each column represents a study participant. Individuals with an Old Brain Age (left panel) have white matter tracts with less integrity (e.g. more purple and blue) compared to those with a Young Brain Age (right panel).

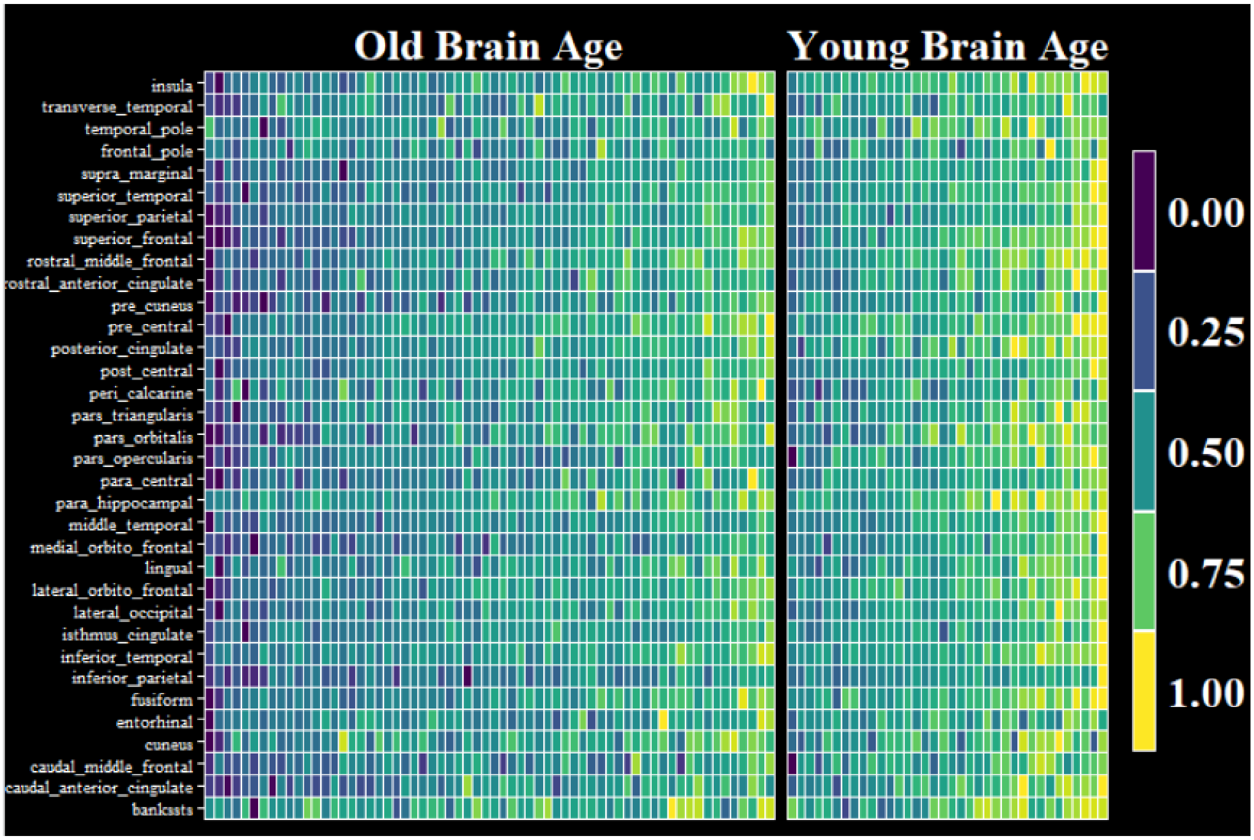

Supplementary Figure 3

Rows are volume and columns are individuals. rh=right hemisphere and lh=left hemisphere. A 0 (purple) is smallest and 1 (yellow) is largest brain volume. Each row represents one volumetric region. Each column represents a study participant. Individuals with an Old Brain Age (left panel) have smaller brain volumes (e.g. more purple and blue) compared to those with a Young Brain Age (right panel).

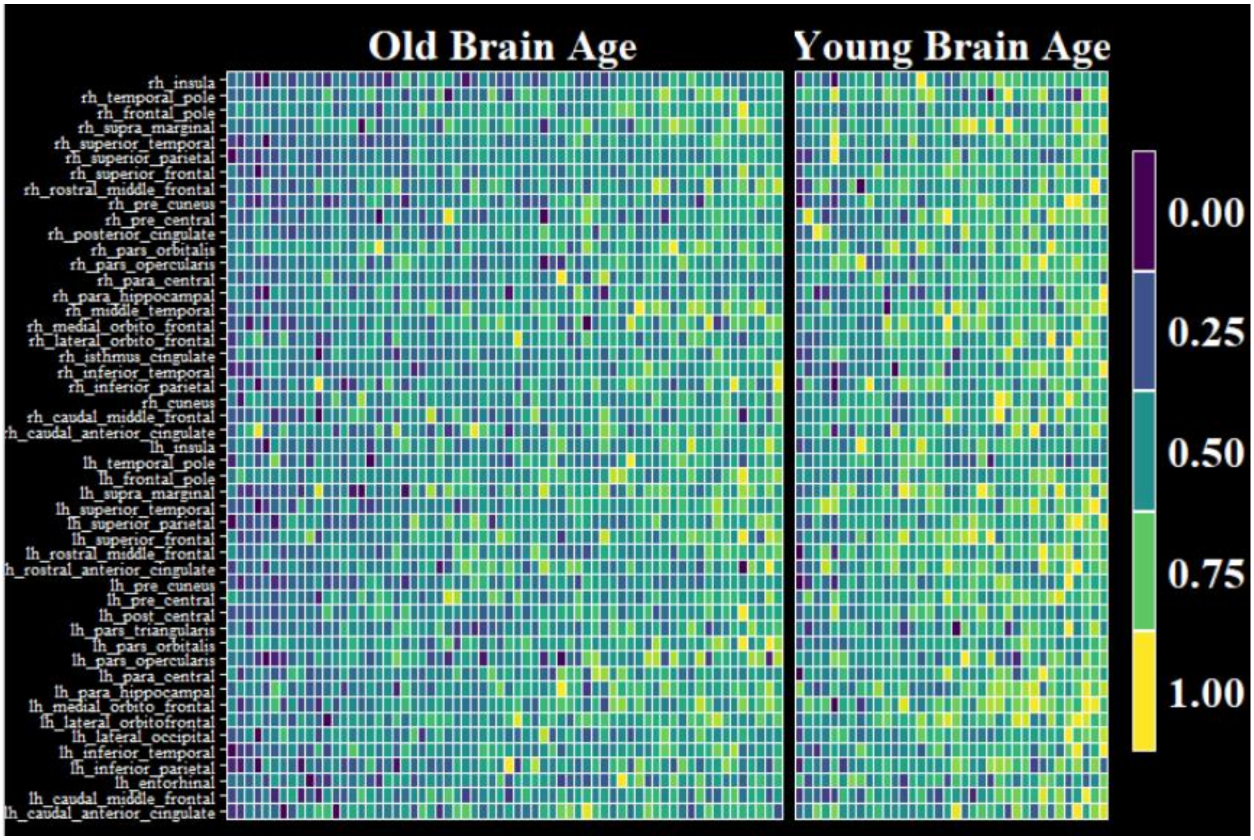

Supplementary Figure 4

Rows are functional connectivity metrics and columns are individuals. A 0 (purple) is weaker and 1 (yellow) is stronger brain connectivity. Each row represents a different network and metric. Each column represents a study participant. Individuals with an Old Brain Age (left panel) have less efficient brains than those with a Young Brain Age (right panel).

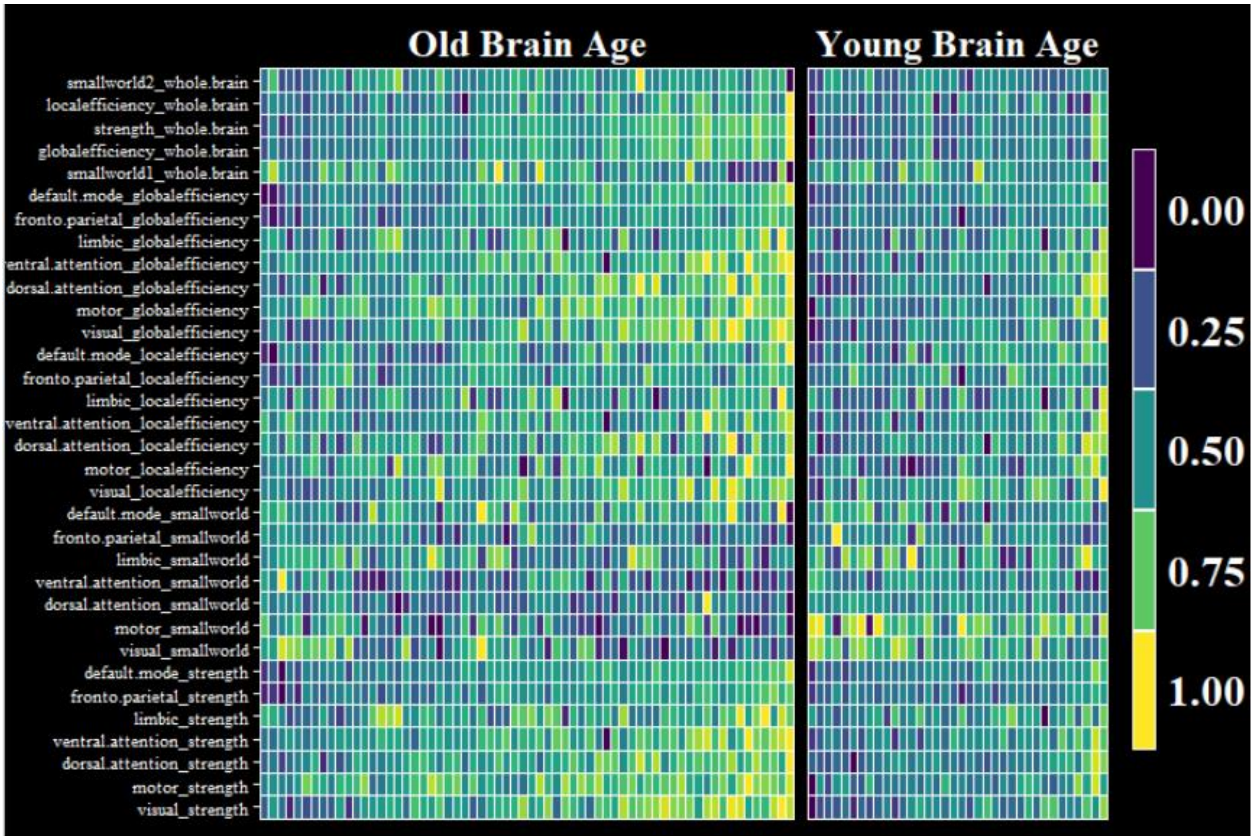

Supplementary Table 1

Measure	Accelerated Brain Aging	Delayed Brain Aging	Δ Aging
DKEFS_Trails.5	0.55	0.52	-0.03
DKEFS_Trails.Errors	0.75	0.73	-0.01
DKEFS_Trails.4.1	0.44	0.45	0.01
DKEFS_Trails.4.23	0.46	0.48	0.02
WASI_VCI	0.53	0.57	0.03
WMS_VMI	0.48	0.52	0.04
DKEFS_Trails.4.5	0.39	0.45	0.05
WMS_AMI	0.49	0.54	0.06
DKEFS_Trails.Composite	0.50	0.56	0.06
WMS_DMI	0.50	0.56	0.06
DKEFS_Trails.4	0.51	0.58	0.07
WMS_IMI	0.47	0.54	0.07
DKEFS_Trails.1	0.56	0.63	0.07
WASI_FSIQ4	0.45	0.55	0.09
WASI_PRI	0.47	0.59	0.12

Average cognitive test scores for the Accelerated and Delayed Brain Aging groups. All test scores are scaled between 0 and 1. The 'Δ Aging' column is derived by subtracting Accelerated Brain Aging from Delayed Brain Aging each cognitive test. The rows in the table are ordered from the smallest to the largest and this corresponds to the ordering in Fig. 2 of the manuscript (left to right on the x-axis).

Supplementary Table 2

Nutrient Category	Nutrient	Accelerated Brain Aging	Delayed Brain Aging	Δ Aging
MUFAs	C20.1n.9	0.45	0.53	0.08
	C18.1n.7	0.50	0.58	0.08
ω-3 PUFAS	C18.3n.3	0.52	0.57	0.05
	C20.5n.3	0.49	0.54	0.05
ω-6 PUFAS	C20.2n.6	0.47	0.52	0.05
	C22.2n.6	0.45	0.52	0.07
Saturated Fatty Acid	C24.0	0.50	0.57	0.07
Antioxidants and Carotenoids	cis-lutein	0.48	0.54	0.06
	trans-lutein	0.50	0.55	0.04
	zeaxanthin	0.45	0.49	0.04
Vitamins	α-tocopherol	0.48	0.53	0.05
	γ-tocopherol	0.46	0.52	0.05
	choline	0.46	0.52	0.05

Nutrient biomarker values scaled between [0,1] for Accelerated and Delayed Aging, along with the difference in magnitude between the two groups (Δ Aging).

Supplementary Table 3

	Coefficient of Variation
C18.1n.7	0.33
C20.1n.9	0.34
C18.3n.3	0.30
C20.5n.3	0.28
C22.2n.6	0.26
C20.2n.6	0.34
C24.0	0.33
cis lutein	0.24
trans lutein	0.26
zeaxanthin	0.27
gamma tocopherol	0.28
alpha tocopherol	0.30
choline	0.28
Intraclass Coefficient Correlation	0.79

Coefficient of variation and intraclass correlation coefficient.

Supplementary Table 4

Domain	Measure	Variables	Accelerated Brain Aging	Delayed Brain Aging	p-value
Demographic	Sex (% Female)	1	64%	61%	0.94
Demographic	Education	1	4.6	4.7	0.24
Demographic	Income	1	4.4	4.8	0.21
Anthropometric	Weight	1	158	163	0.36
Anthropometric	Height	1	65.7	66.5	0.36
Anthropometric	Waist	1	36.0	35.4	0.70
Anthropometric	Hip	1	40.2	40.6	0.65
Fitness	Body Mass Index	1	26.0	26.0	0.70
Fitness	Resting Heart Rate (RHR)	1	68.7	66.4	0.27
Fitness	Physical Activity Scale (PAS)	1	1.96	1.73	0.30
Fitness	Metabolic Equivalent of VO2 (MET)	1	8.0	7.7	0.46

Demographic, Anthropometric and Fitness covariates collected in the study do not differ between Accelerated and Delayed Brain Aging. The p-value is from a t-test comparing the means of Accelerated and Delayed Brain Aging for a covariate. Education was measured on a 5-point scale, where a 5 is at least an undergraduate degree. Income was measured on a 5-point scale, where 5 is >$100K). Height, Waist and Hip variables were all measured in inches. Resting Heart Rate was measured in beats/minute.

Supplementary Table 5

Nutrient	r	p-value
choline	-0.12	0.24
alpha_tocopherol	-0.02	0.78
C18.3n.3	0.03	0.70
C20.5n.3	0.09	0.29
zeaxanthin	0.10	0.24
gamma_tocopherol	0.13	0.14

Correlation Between Biomarkers and Diet Questionnaire
