# Supplementary material for: Investigating nutrient biomarkers of healthy brain aging: a multimodal brain imaging study
Source: NPJ Aging. 2024 May 21;10(1):27. doi: 10.1038/s41514-024-00150-8 (PMC11109270; doi:10.1038/s41514-024-00150-8)
Supplement: Supplementary file 2 — Reporting Summary [file 41514_2024_150_MOESM2_ESM.pdf]

nature portfolio

Corresponding author(s): Aron K. Barbey

Last updated by author(s): Mar 29, 2024

# Reporting Summary

Nature Portfolio wishes to improve the reproducibility of the work that we publish. This form provides structure for consistency and transparency in reporting. For further information on Nature Portfolio policies, see our Editorial Policies and the Editorial Policy Checklist.

## Statistics

For all statistical analyses, confirm that the following items are present in the figure legend, table legend, main text, or Methods section.

| n/a | Confirmed | |
|---|---|---|
| ☐ | ☒ | The exact sample size (*n*) for each experimental group/condition, given as a discrete number and unit of measurement |
| ☐ | ☒ | A statement on whether measurements were taken from distinct samples or whether the same sample was measured repeatedly |
| ☐ | ☒ | The statistical test(s) used AND whether they are one- or two-sided<br>*Only common tests should be described solely by name; describe more complex techniques in the Methods section.* |
| ☐ | ☒ | A description of all covariates tested |
| ☐ | ☒ | A description of any assumptions or corrections, such as tests of normality and adjustment for multiple comparisons |
| ☐ | ☒ | A full description of the statistical parameters including central tendency (e.g. means) or other basic estimates (e.g. regression coefficient) AND variation (e.g. standard deviation) or associated estimates of uncertainty (e.g. confidence intervals) |
| ☐ | ☒ | For null hypothesis testing, the test statistic (e.g. *F*, *t*, *r*) with confidence intervals, effect sizes, degrees of freedom and *P* value noted<br>*Give P values as exact values whenever suitable.* |
| ☒ | ☐ | For Bayesian analysis, information on the choice of priors and Markov chain Monte Carlo settings |
| ☒ | ☐ | For hierarchical and complex designs, identification of the appropriate level for tests and full reporting of outcomes |
| ☐ | ☒ | Estimates of effect sizes (e.g. Cohen's *d*, Pearson's *r*), indicating how they were calculated |

*Our web collection on statistics for biologists contains articles on many of the points above.*

## Software and code

Policy information about availability of computer code

| | |
|---|---|
| Data collection | *Provide a description of all commercial, open source and custom code used to collect the data in this study, specifying the version used OR state that no software was used.* |
| Data analysis | R Statistical Software |

For manuscripts utilizing custom algorithms or software that are central to the research but not yet described in published literature, software must be made available to editors and reviewers. We strongly encourage code deposition in a community repository (e.g. GitHub). See the Nature Portfolio guidelines for submitting code & software for further information.

## Data

Policy information about availability of data

All manuscripts must include a data availability statement. This statement should provide the following information, where applicable:

- Accession codes, unique identifiers, or web links for publicly available datasets
- A description of any restrictions on data availability
- For clinical datasets or third party data, please ensure that the statement adheres to our policy

The individual de-identified participant data can be made available upon request.

## Research involving human participants, their data, or biological material

Policy information about studies with human participants or human data. See also policy information about sex, gender (identity/presentation), and sexual orientation and race, ethnicity and racism.

| | |
|---|---|
| Reporting on sex and gender | Sex was included as a covariate in all analyses. Sex was tested as a covariate that accounted for group differences but was not significant. |
| Reporting on race, ethnicity, or other socially relevant groupings | All participants were Caucasian and this is included in both the population description and study limitations, as the effect of nutrition may differ by race or ethnicity. |
| Population characteristics | Sex, age, BMI, education and income were included in this study as covariates. |
| Recruitment | Participants were recruited through Carle hospital and the University of Illinois. |
| Ethics oversight | University of Illinois IRB |

Note that full information on the approval of the study protocol must also be provided in the manuscript.

# Field-specific reporting

Please select the one below that is the best fit for your research. If you are not sure, read the appropriate sections before making your selection.

☐ Life sciences ☒ Behavioural & social sciences ☐ Ecological, evolutionary & environmental sciences

For a reference copy of the document with all sections, see nature.com/documents/nr-reporting-summary-flat.pdf

# Behavioural & social sciences study design

All studies must disclose on these points even when the disclosure is negative.

| | |
|---|---|
| Study description | The study is a cross-sectional design with quantitative data. |
| Research sample | This cross-sectional study enrolled 100 healthy elderly adults from the Illinois Brain Aging Study cohort, a sample of community-dwelling Caucasian men and women aged 65-75 years. Participants were neurologically healthy and did not have evidence of cognitive impairment, as determined by a score of lower than 26 on the Mini-Mental State Examination (40). |
| Sampling strategy | Participants were recruited to assess the effects of nutrition on healthy brain and cognitive aging. Data for 100 participants was collected because this represents a statistically high-powered study for brain imaging analyses. |
| Data collection | Complete data collection details are included in the supplemental materials. Briefly, cognitive testing was completed by paper-and-pencil tests in the presence of a test administrator; fMRI acquistion was in the University of Illinois Beckman Institute imaging lab and a fMRI technician and lab personnel were present during the scan; and hospital nurses at Carle hospital collected the blood biospecimens for the nutritional assays. |
| Timing | May. 2014-May 2016 |
| Data exclusions | All participants with valid fMRI scans were included in the study. |
| Non-participation | 29 participants enrolled but did not complete 1 or more of the study sessions. |
| Randomization | No randomization was employed. |

# Reporting for specific materials, systems and methods

We require information from authors about some types of materials, experimental systems and methods used in many studies. Here, indicate whether each material, system or method listed is relevant to your study. If you are not sure if a list item applies to your research, read the appropriate section before selecting a response.

## Materials & experimental systems

| n/a | Involved in the study |
|---|---|
| ☒ | ☐ Antibodies |
| ☒ | ☐ Eukaryotic cell lines |
| ☒ | ☐ Palaeontology and archaeology |
| ☒ | ☐ Animals and other organisms |
| ☒ | ☐ Clinical data |
| ☒ | ☐ Dual use research of concern |
| ☒ | ☐ Plants |

## Methods

| n/a | Involved in the study |
|---|---|
| ☒ | ☐ ChIP-seq |
| ☒ | ☐ Flow cytometry |
| ☐ | ☒ MRI-based neuroimaging |

# Plants

Seed stocks

Not applicable to our study.

Novel plant genotypes

Not applicable to our study.

Authentication

Not applicable to our study.

# Magnetic resonance imaging

## Experimental design

Design type

Resting state.

Design specifications

Not applicable.

Behavioral performance measures

Behavioral measures were not collected concurrent with scanning. Cognitive testing was completed in a separate session.

## Acquisition

Imaging type(s)

Functional and structural.

Field strength

3 Tesla

Sequence & imaging parameters

A high-resolution multi-echo T1-weighted magnetization prepared gradient-echo structural image was acquired for each participant (0.9 mm isotropic, TR: 1900 ms, TI: 900 ms, TE = 2.32 ms, with GRAPPA and an acceleration factor of 2). The functional neuroimaging data were acquired using an accelerated gradient-echo echoplanar imaging (EPI) sequence sensitive to blood oxygenation level dependent (BOLD) contrast (2.5 x 2.5 x 3.0 mm voxel size, 38 slices with 10% slice gap, TR = 2000 ms, TE = 25 ms, FOV = 230 mm, 90-degree flip angle, 7 minute acquisition time).

Area of acquisition

Whole brain.

Diffusion MRI ☒ Used ☐ Not used

Parameters

Whole brain diffusion tensor imaging was acquired with the following parameters: FOV = 240 x 240 mm; 72 slices, slice thickness = 2 mm; TE = 98 ms; TR = 10,000 ms; in-plane resolution = 1.875 x 1.875 mm; diffusion encoding directions = 30; b = 0 s/mm2 and 1,000 s/mm2. Data were processed using the University of Oxford's Center for Functional Magnetic Resonance Imaging of the Brain (FMRIB) Software Library (FSL) release 5.0 diffusion toolbox (FDT). Eddy current correction was accomplished using the eddy correction tool and a diffusion tensor model was fit in each voxel using the DTIFIT tool, which generates fractional anisotropy (FA) values in every voxel. FA images were further processed using the FSL tract-based spatial statistics (TBSS) toolbox, which projects each subjects' FA data onto a mean white matter skeleton, representing the white-matter tracts common to all subjects. Mean FA within the white matter skeleton for specific regions of interest were calculated for each subject using the JHU ICBM DTI-81 atlas.

## Preprocessing

Preprocessing software

All MRI data processing was performed using FSL tools available in FMRIB Software Library version 5.0. The high-resolution T1 Magnetization-Prepared Rapid Gradient-Echo (MPRAGE) was brain extracted using the Brain Extraction Tool (BET). FMRIB's Automated Segmentation Tool (FAST) was applied to delineate gray matter, white matter, and cerebral spinal fluid (CSF)

voxels.

Normalization

The resting-state fMRI data were pre-processed using the FSL FMRI Preprocessing and Model-Based Analysis (FEAT) analysis tool. Pre-processing entailed: slice timing correction, motion correction, spatial smoothing (3 mm full width at half maximum kernel), nuisance signal regression (described below), standard fMRI temporal bandpass filtering (0.009 - 0.1 Hz, linear registration of functional images to structural images, and non-linear registration of structural images to the MNI152 brain template (2 mm isotropic voxel resolution).

Normalization template

MNI152

Noise and artifact removal

Nuisance variables were modeled via General Linear Modeling (GLM) analyses to remove spurious correlations, noise introduced by head motion, and variables of no interest. These included head motion correction parameters (using the extended 12 motion parameters estimated in FEAT preprocessing), modeling of individual volume motion outliers estimated using DVARS (outliers flagged using the boxplot cutoff 1.5 x interquartile range), and averaging of mean white matter and cerebrospinal fluid signals across all voxels identified from the segmentation of the high resolution MPRAGE. The fully preprocessed resting-state fMRI data comprised the residual obtained from fitting these nuisance variables in the GLM framework. The residuals were transformed into normalized MNI152 space and re-sampled to 4 mm isotropic voxels to reduce computational complexity in post data processing for network analysis.

Volume censoring

Cortical reconstruction was performed with the Freesurfer image analysis software, http://surfer.nmr.mgh.harvard.edu/. For this analysis, all the cortical gray matter volumes provided by the Freesurfer parcellation were examined. This included 68 regions throughout the frontal, parietal, temporal, and occipital lobes. In line with current practice volumetric measures were adjusted for intracranial volume and sex using a regression model. The adjusted values were then used in further statistical analyses.

## Statistical modeling & inference

Model type and settings

Once the functional and structural brain measures were determined, they were modeled jointly in hierarchical clustering.

Effect(s) tested

Not applicable.

Specify type of analysis: [x] Whole brain [ ] ROI-based [ ] Both

Statistic type for inference
(See Eklund et al. 2016)

All brain measures were simultaneously included in a hierarchical clustering model.

Correction

Not applicable, as all brain measures were included simultaneously in a single hierarchical clustering model.

## Models & analysis

| n/a | Involved in the study |
|---|---|
| [ ] | [x] Functional and/or effective connectivity |
| [ ] | [x] Graph analysis |
| [x] | [ ] Multivariate modeling or predictive analysis |

Functional and/or effective connectivity

The efficiency of brain network function was examined by investigating the small-world organization of 7 well-established intrinsic connectivity networks of the brain 46 (Figure 1). A small-word organization represents the optimal balance of local and global network efficiency, providing a parsimonious neural architecture that supports high local clustering (local efficiency) and short average path length (global efficiency). The procedure for computing small-world propensity is presented below.
First, the mean fMRI BOLD time series was extracted from subjects' grey matter voxels using the Craddock parcellated brain atlas as a mask. This parcellation of 200 regions provided whole brain coverage and sufficiently high spatial resolution for conducting network analysis. A subject-wise functional connectivity matrix reflecting pairwise Pearson correlations between the mean BOLD time series signals obtained from nodes defined by the Craddock atlas was then computed and Fisher's Z-transformed to achieve normality. These were standardized to Z-scores through multiplication with their standard deviation approximated as $\sigma = 1/\sqrt{(n-3)}$, where n is the number of time points corresponding to the BOLD signal. A Bonferroni-corrected statistical Z-threshold was applied to identify significant positive correlations ($p<0.05$) within each subject's whole brain functional connectivity matrix derived from Craddock's 800 parcellation atlas. The thresholded Z-scores were rescaled to represent connection weights ranging from 0 to 1. Based on these positive connection weights, weighted connectivity matrices representing functional connectivity between nodes representative of seven intrinsic connectivity networks were obtained for each subject. The seven intrinsic connectivity network maps—visual, somatosensory, limbic, default mode, dorsal attention, ventral attention and frontoparietal—are at https://surfer.nmr.mgh.harvard.edu/fswiki/CorticalParcellation_Yeo2011.

Graph analysis

We then examined small-world propensity within the rescaled connectivity matrices derived for each of the seven intrinsic connectivity networks. Small world propensity Φ is calculated as the fractional deviation between a network's clustering coefficient, Cobs, and characteristic path length, Lobs, from both lattice (Clatt, Llatt) and random (Crand, Lrand) networks constructed with the same number of nodes and the same degree distribution107:
$\phi=1-\sqrt{((\Delta\_C^2+\Delta\_L^2)/2)}$ , (1)

where,
$\Delta\_C=$ (C_latt- C_obs)/(C_latt- C_rand ) , (2)
and
$\Delta\_L=$ (L_obs- L_rand)/(L_latt- L_rand ) . (3)
The ratios ΔC and ΔL represent the fractional deviation of the metric (Cobs or Lobs) from its respective null model (a lattice or random network).
